# Strengthening research preparedness for crises: lessons from Norwegian government agencies in using randomized trials and quasi-experimental methods to evaluate public policy interventions

**DOI:** 10.1186/s12961-024-01271-y

**Published:** 2025-01-13

**Authors:** Unni Gopinathan, Ingeborg Elgersma, Therese Dalsbø, Mona Bjørbæk, Atle Fretheim

**Affiliations:** 1 Centre for Epidemic Interventions Research, Norwegian Institute of Public Health, Oslo, Norway; 2 National Institute of Occupational Health, Oslo, Norway; 3 Faculty of Health Sciences, Oslo Metropolitan University, Oslo, Norway

**Keywords:** RCTs, Policy experiments, Quasi-experiments, Evidence-informed policymaking, Research ethics, Politics

## Abstract

During public health crises such as pandemics, governments must rapidly adopt and implement wide-reaching policies and programs (“public policy interventions”). A key takeaway from the coronavirus disease 2019 (COVID-19) pandemic was that although numerous randomized controlled trials (RCTs) focussed on drugs and vaccines, few policy experiments were conducted to evaluate effects of public policy interventions across various sectors on viral transmission and other consequences. Moreover, many quasi-experimental studies were of spurious quality, thus proving unhelpful for informing public policy. The pandemic highlighted the need to continuously develop competence, capacity and a robust legal–ethical foundation for impact evaluations well before crises occur. It raised a crucial question: how can governments in non-crisis times develop capabilities to generate evidence on the effects of public policy interventions, thus enabling a rapid and effective research response during public health crises? We conducted a mapping to explore how government agencies in Norway use RCTs and quasi-experimental methods to strengthen the evidence base for public policy interventions and to identify barriers and facilitators to their use. Contributing to the study were 10 government agencies across sectors such as development assistance, education, health, social welfare, statistics and taxation. Many of these agencies have conducted or commissioned RCTs or quasi-experimental studies in the past 5 years, with evaluations ranging from 1 or 2 to more than 15 per agency. The measures evaluated included organizational, educational and financial interventions and interventions for oversight and sanctions. Some agencies have internal capabilities for designing and conducting evaluations, while others commissioned such studies to universities and other research institutions. Agencies reported examples where enhanced communication among implementers, researchers, ministries and political leaders facilitated impact evaluations, and these lessons offer opportunities for cross-sector knowledge-sharing to help strengthen rigorous evaluations of public policy interventions. Despite their potential, various government agencies report that randomized and quasi-experimental studies face legal, ethical, political and practical barriers that affect their use. For instance, the urgency of politicians to implement policies at scale has led to the discontinuation of trials and hindered learning from their effects. The surveyed agencies stressed the importance of legislation providing clear guidelines on when differential treatment can be justified and when informed consent requirements can be waived, as well as faster and clearer processes for managing privacy concerns related to data access. Crucially, greater political acceptance for systematically and gradually implementing reforms, including using randomization, could strengthen evidence-informed public policy, enhancing the scaling-up of effective interventions and deprioritizing ineffective ones.

**Supplementary Information:**

The online version contains supplementary material available at 10.1186/s12961-024-01271-y.

## Background

During public health crises such as pandemics and other health threats governments must rapidly adopt and implement policies and programs (“public policy interventions”) that impact millions of people. Cutting across sectors ranging from health to education, labour, social services and transportation, many of these interventions are characterized by uncertainty about their benefits and costs, emphasizing the need for a prompt and rigorous research response to generate the evidence required to manage uncertainty and guide government actions.

Over the past two decades, there has been growing focus on evidence-informed policy supported by randomized controlled trials (RCTs) and quasi-experimental methods to evaluate public policy [1, 2]. RCTs are commonly used to evaluate the efficacy of medical interventions such as drugs and vaccines, whereas their application in assessing broader, population-level policies and interventions has been less prominent. However, led by major research centres such as the Abdul Latif Jameel Poverty Action Lab (J-PAL) and the International Initiative for Impact Evaluation, there has been increasing use of RCTs to inform public policy in areas such as poverty reduction, health and education [1]. Another term for RCTs of public policy interventions is "policy experiments" [3]. In cases where policy experiments are infeasible, due to legal, ethical, political or practical reasons, quasi-experimental evaluations can be another reliable source of evidence, provided that the necessary data sources are available and the public policy intervention has been implemented in a manner that aligns with the key assumptions of these methods [4, 5].

A key takeaway from the coronavirus disease 2019 (COVID-19) pandemic was that while numerous RCTs were conducted on drugs and vaccines, relatively few policy experiments were conducted to evaluate broader public policy interventions (also referred to as public health and social measures) implemented across various sectors to prevent coronavirus transmission [6–8]. Moreover, many quasi-experimental studies proved to be of spurious quality and unhelpful for informing public policy [9]. These lessons highlighted the need for greater investment in research preparedness and stronger scientific capabilities across government sectors to better generate timely and reliable evidence during public health crises.

In Norway, the absence of rigorously generated evidence about the benefits and costs of public health and social measures repeatedly sparked public debate, involving discussions between the government and scientific community [10]. The question of how to build an improved knowledge system for managing crises has been given cross-departmental attention through a body coordinated by the Ministry of Education and including representatives from various other ministries [11]. In 2022, this body commissioned two expert group reports on the following themes: (1) effective and secure infrastructure for access to, sharing and use of relevant statistics and data in crises and (2) legal and ethical issues related to the collection, availability, sharing and use of data, as well as the use of randomized trials, in crises. Important lessons from these reports were that competence and capacity, along with a clear legal and ethical foundation for impact evaluations, should be continuously developed before crises occur [12].

A crucial step in strengthening research preparedness for public health crises is therefore to assess the experiences of government agencies with using RCTs and quasi-experimental methods for evaluating public policy during non-crisis periods. Internationally, few assessments of government agencies’ experiences with these approaches exist. One report by J-PAL, examining state and local governments in the United States, highlights opportunities and barriers to using randomized rollout as an evaluation strategy [13]. An opportunity to address this gap in knowledge about the barriers and facilitators to governmental use of RCTs and quasi-experimental methods arose when the Norwegian Ministry of Health and Care Services commissioned the Norwegian Institute of Public Health (NIPH) in 2023 to undertake a mapping. NIPH explored how government agencies in Norway use these methods to strengthen the evidence base for public policy decisions. This assessment provides insights into the Norwegian state administration’s knowledge base and prerequisites, as well as key challenges and opportunities for using RCTs and quasi-experimental methods to inform policy with evidence. Exploring these experiences uncovered key barriers and opportunities to enhance research preparedness for times of crisis, which could be relevant to other countries as well.

## Methods

### Context and sampling

The objective of the mapping was to coordinate a survey of relevant government agencies and their experience with RCTs and quasi-experimental methods for evaluating public policy interventions. In addition to the NIPH, five other government agencies were instructed to contribute to the mapping in their respective Letters of Allocation – which stipulates the resources and tasks assigned to each government agency – sent by their respective ministries. These were: the Norwegian Labour and Welfare Administration; the Norwegian Tax Administration; the Directorate for Children, Youth and Family Affairs; the Directorate for Education and Training; and the Norwegian Agency for Development Cooperation (Norad). These were known to have experience with either conducting impact evaluations or commissioning such studies, and were the primary contributors to the mapping.

In the assignment from the MoH, it was also specified that the mapping could, if relevant, include experiences from other government agencies or municipalities. To gain additional insights, we purposefully sampled the following agencies on the basis of recommendations from the primary agencies or by identifying them as contributors to impact evaluations identified during the mapping: the Norwegian Directorate for Higher Education and Skills; Statistics Norway; the Norwegian Directorate for Health; and Norwegian State Educational Loan Fund.

We also reached out to the research and innovation coordinators of the municipalities of Bergen and Trondheim. For Oslo, we did not identify a suitable focal point to engage with. In Trondheim, the focal point was unable to provide examples of impact evaluations of municipal policies. The focal point in Bergen provided an overview of researcher-initiated projects, including RCTs, in which municipal staff acted as observers or participants. However, these were not RCTs of public policy interventions initiated by the municipality, and were therefore considered outside the scope of this mapping.

### Survey

We designed a survey in Norwegian to collect experiences with RCTs or quasi-experimental evaluations (Appendix 1, translated from Norwegian to English). The survey was organized into four parts: (1) key definitions, including of “public policy interventions”, “randomized trials” and “quasi-experimental studies”; (2) questions about the use of RCTs; (3) questions about the use of quasi-experimental studies; and (4) general questions about capacity and pre-requisites for conducting impact evaluations. Except for a question about the approximate number of RCTs or quasi-experimental evaluations in the past 5 years, all other queries elicited qualitative responses. The questions were not specifically focussed on RCTs or quasi-experimental evaluations conducted during the COVID-19 pandemic or other public health crises, although these evaluations could also be included. Before designing the survey, we held an initial 30-min meeting with the focal points at each participating agency to discuss the survey’s scope and gain insights into their experience with RCTs and quasi-experimental methods. This information was used to inform the relevant questions that could be included. A draft version of the survey was shared to check whether the questions were similarly understood across the agencies to improve the clarity of the questions and to include missing questions. The survey was sent on 2 March 2023, and 3 weeks were allowed for the response. The participants could respond using a digital survey designed in KoboToolbox (https://www.kobotoolbox.org/) or return a Word file with responses.

### Analysis

The primary author read and compared all the survey responses, analysing and distilling a summary of key facilitators and barriers that were common across government agencies. Additionally, specific barriers unique to one or a few agencies were noted. Principles of thematic analysis, including familiarizing with the text, labelling text fragments with codes and organizing these under major and minor themes were used to analyse the survey responses [14]. The questions served as the framework for organizing these findings, addressing two main aspects. Descriptively, we focussed on the quantity of RCTs and how each agency was organized to conduct or commission evaluations. Analytically, we focussed on ethical, legal, political, practical and other factors influencing the extent RCTs or quasi-experimental evaluations were used. These findings were then discussed with the authors before the initial draft was prepared. This draft was circulated among contributors from the primary government agencies. After a first round of inputs, a second draft was circulated for a final round of comments before the main findings were defined.

## Main findings

### Descriptive overview and organization

Three government agencies (NIPH; Norwegian Labour and Welfare Administration; Norwegian Tax Administration) each reported more than 15 RCTs over the past 5 years, while Directorate for Education and Training; Directorate for Children, Youth and Family Affairs; and Norad reported to have commissioned between 1 and 5. On quasi-experimental evaluations, NIPH and Norwegian Labour and Welfare Administration each reported more than 15 evaluations over the past 5 years, the Norwegian Tax Administration reported to have performed between 6 and 15, and Directorate for Education and Training and Norad reported between 1 and 5.

These agencies were organized differently with respect to conducting RCTs and quasi-experimental evaluations. NIPH Norwegian Labour and Welfare Administration and Norwegian Tax Administration primarily design and conduct such evaluations themselves with their own expertise and capacity, while the rest commissioned such studies. Tables 1 and 2 provide examples of public policy interventions that have been evaluated with RCTs or quasi-experimental designs.

**Table 1:** Examples of randomized trials conducted or commissioned by government agencies

Agency	Sector	Public policy intervention	Stakeholders involved
Norwegian Institute of Public Health	Health	Extended opening hours at the government-owned alcoholic beverage retailer	The Norwegian Monopoly for Wine and Spirits, Ministry of Health
The Norwegian Directorate for Education and Training	Education	Adding half-time public health nurse positions to schools	Ministry of Education, Norwegian Centre for Learning Environment and Behavioural Research in Education, Norwegian Institute of Public Health
The Norwegian Directorate for Children, Youth and Family Affairs	Early childhood development	Nurse–family partnership: a nurse home visiting program to provide support and guidance to first-time mothers during pregnancy and until the child’s second birthday	Municipalities, several academic institutions in Norway
The Norwegian Labour and Welfare Administration	Immigration and labour market	Individual Placement and Support programmes offered to increase labour market participation among immigrants	Bergen municipality
Norwegian Tax Authority	Taxation	Using nudging in a digital tax return to increase compliance	
Norwegian Agency for Development Cooperation	International development	Results-based financing of health services	Governments, health systems and academic institutions in low- and middle-income countries

**Table 2:** Examples of quasi-experimental evaluations conducted or commissioned by government agencies

Agency	Sector	Public policy intervention
Norwegian Tax Authority	Taxation	Effects of oversight on tax compliance
Norwegian Agency for Development Cooperation	International development	Norway–India Partnership Initiative effect on maternal and child health
Norwegian Institute of Public Heath	Public health	Effects of standardized packaging of tobacco products on tobacco use
The Norwegian Directorate for Education and Training	Education	Effects of free preschool on school performance of children
The Norwegian Labour and Welfare Administration	Labour market	Effects of shorter duration of work assessment allowance on the eligibility for disability insurance and labour supply

### Factors influencing the conduct of randomized trials and quasi-experimental evaluations

We present factors influencing the conduct of randomized and quasi-experimental evaluations as reported by the different agencies. The factors are presented according to the pre-defined categories used in the survey: methodological, legal, ethical, political and practical factors, as well as a group of “other” factors that influenced the conduct of impact evaluations. For specific examples, we explore how these factors acted as barriers to running planned RCTs and in some cases also preventing quasi-experimental evaluations. However, these factors are not permanent obstacles; by addressing them through new legislation, clearer guidance on managing ethical trade-offs or building increased political support for impact evaluations, better conditions can be created for generating evidence that is useful to policymakers. We explore such measures in the second part of this section, under Main opportunities.

#### Methodological challenges

Respondents noted that a key methodological challenge with RCTs is that the transferability of results is not always assured and depends heavily on the specific context of the study. The agencies have also experienced that the effect of an intervention aimed at groups (e.g. schools or municipalities) may “leak” between the intervention and control groups, and thus produce misleading results. Respondents pointed out that a primary methodological limitation of quasi-experimental studies is the risk of biased results arising from differences between groups that are unrelated to the intervention being studied.

For many randomized studies, achieving sufficient statistical power is a significant challenge. Studies examining interventions targeting groups of individuals (e.g. welfare offices, schools, health clinics or municipalities) have faced difficulties in recruiting a sufficient number of groups to randomize. For example, when NIPH proposed evaluating the effects of reopening schools during the early phase of the COVID-19 pandemic, it was estimated that all schools in the country would need to be included in the randomization to achieve sample sizes large enough to draw reliable conclusions. Similarly, the Norwegian Directorate for Children, Youth and Family Affairs described how the target group for an intervention, such as specific user groups of child welfare services, may be so limited that recruiting enough participants to achieve sufficient statistical power can be challenging.

#### Legal tensions

A central challenge to conducting cluster-randomized trials is the informed consent requirement, which can make many population-level studies infeasible. When comparing the experiences of different government agencies, there was a clear difference between interventions regulated by Norway’s Health Research Act and those governed by other laws. Under the Health Research Act, the common interpretation has been that informed consent is an absolute requirement, even for trials of population-level interventions. It is less clear when consent is required for studies subject to other legislation and research ethics guidelines. Several agencies reported that their assessment of the need for consent depends on the type of intervention under evaluation, such as how intrusive it is for the study participants. A second legal tension is that differential treatment of subjects, a key requirement for conducting impact evaluations, may conflict with principles of equal treatment and legal provisions safeguarding individuals’ fundamental rights and services. For instance, under the National Insurance Act, evaluations of the Norwegian Labour and Welfare Administration's benefits cannot involve the restriction of individuals’ rights or impose greater obligations than what is stipulated by the law. In practice, this means that individuals eligible for benefits cannot be subjected to differential treatment in impact evaluations; therefore, a control group cannot receive less than the status quo.

Another example is from the education sector, where the Education Act and its regulations set forth various minimum requirements to ensure students receive an equitable and high-quality education. In the context of impact evaluations, especially RCTs, this means that interventions must not infringe upon students’ fundamental rights, impose an unreasonable burden or result in substantial disparities in the quality of education provided. However, the law permits the Norwegian Directorate for Education and Training, under the Ministry of Education and Research, to approve deviations from regulations and laws for temporary pedagogical or organizational experiments upon request from municipalities or county municipalities [15].

Some legal tensions are sector specific. Norad’s administration of grants is governed by regulatory frameworks that define the scope of fund utilization. Impact evaluations have previously not been explicitly mentioned, making it difficult to finance RCTs with such funds. However, the national budget 2022–2023 came with an overarching directive calling for evidence-informed development assistance and for impact evaluations to be integrated in programmes [16]. This is expected to provide greater flexibility in financing impact evaluations in the development sector.

Finally, the conduct of impact evaluations may be subject to different legal frameworks, leading to confusion about the legal basis for such assessments. Government agencies reported facing an uncertain legal basis when it comes to conducting RCTs and quasi-experimental studies, including the authority to collect and process personal data about study participants. This uncertainty can impede the planning and execution of thorough impact evaluations. The Norwegian Tax Administration and the Norwegian Labour and Welfare Administration reported to have undergone or to be currently reviewing the legal foundation for conducting impact evaluations, including an assessment of the legal basis for time-limited differential exposure to public policy interventions and the handling of personal data during trials.

#### Political factors

The extent, scale and pace at which public policy interventions are implemented are largely politically driven. Therefore, there is significant political leeway to facilitate policy experiments. The experiences reported by respondents converged on three types of political factors that influence the implementation of RCTs or quasi-experimental evaluations.

The first factor is the need for greater political acceptance that many public policy interventions involve significant uncertainty, and that enabling RCTs or quasi-experimental evaluations can be crucial in reducing this uncertainty. However, this requires greater political support for systematic, time-limited and differential exposure of public policy interventions to enable evaluation of effects.

A second factor is that a mismatch between decision-makers’ desire for quick answers and the time required for conducting rigorous impact evaluations may prevent the use of such evidence. Thorough impact evaluations involve time for summarizing the knowledge base, designing interventions suitable for impact assessment, obtaining ethical approvals and data access and ensuring that the trial period is sufficiently long to measure relevant effects. While there is potential to expedite impact evaluations, for instance, through faster processes for ethical approval and data access, patience among decision-makers is crucial to ensure that timely research findings can inform their decision-making processes. An essential challenge reported across government agencies is that policy-relevant effects often emerge long after individuals have experienced differential exposure to an intervention, such as the impact of a job program on long-term labour market affiliation.

Finally, political processes, such as changes in government or budgets, may inadvertently expose the control group to the intervention being tested, thereby biasing the results of a RCT. A concrete example reported by the Directorate for Education and Training was when the government decided to introduce a new teacher density norm starting in the fall of 2018. This deviated from the idea of a gradual reform since the government did not wait for the results from research initiated two years earlier to evaluate the effects of having more teachers in schools. The Two Teachers project, originally intended to assess outcomes from grades 1 through 4, had to limit its conclusions to the first two grades of primary school due to its reliance on participating schools maintaining stable pupil-teacher ratios[17, 18].

#### Ethical considerations

The survey responses highlighted three ethical considerations for planning and executing impact evaluations, which mostly apply to RCTs. First, when an intervention is expected to provide a benefit to individuals or groups, some perceive that it is unethical to withhold the intervention from a random sample of the population. For example, such ethical concerns may arise in trials of poverty-reduction measures, when the tested intervention is not provided to impoverished and vulnerable populations.

Second, randomized studies can be hard to justify when there is a risk for negative consequences for participants, especially for children and youth. For example, when children were randomised to small-group maths instruction within classes, parents pointed to the risk of demotivation for the children participating in the control group not receiving extra maths teaching. The National Research Ethics Committee for Social Sciences and Humanities highlighted several issues related to these types of studies, including the need for child expertise in research teams and careful ethical justification, considering their vulnerability, needs, rights and special circumstances[19]. For vulnerable children, the Norwegian Directorate for Children, Youth and Family Affairs reported challenges with obtaining consent from both parents and unclear reporting processes for managing critical incidents.

A third consideration concerns whether and how informed consent should be obtained. While it is ethically questionable to not inform participants about their involvement in a RCT, requiring consent from all participants makes it near to impossible to evaluate group-level interventions. The norm in health research is that consent must be obtained when research is conducted on humans, human biological material or health data, as outlined in the aforementioned Health Research Act [20]. The NIPH reported several planned RCTs on infection control which could not be conducted because obtaining individual consent was practically impossible.

#### Other challenges

RCTs and similar experimental interventions often encounter scepticism and resistance, partly due to the perception that one group is denied the benefits of the intervention as part of the experiment. For example, the Nurse Family Partnership program funded by the The Norwegian Directorate for Children, Youth and Family Affairs faced resistance because the control group, consisting of young pregnant women, would not receive access to the program. Such concerns highlight the importance of obtaining community and service buy-in to enhance the feasibility of RCTs.

Another challenge with RCTs is the potential disruptions to regular delivery of services, for example in health centres or schools. Participating institutions may need to devote extra time and human resources to provide additional services or enable data collection processes. In some cases, existing infrastructure for delivering public services can be used as part of the randomized experiment. One example is when the Norwegian State Educational Loan Fund infrastructure was used to randomize stipend pay-outs for a trial of incentives for professional development of skilled workers (Box 1).

The cost of RCTs was also cited as a major challenge, since these often consume a significant portion of the total allocation available for research programs. The cost of conducting an RCT varies widely. The studies identified and described in the survey have incurred estimated costs ranging from 90 000 to 3 million euros. The size is influenced by whether there are substantial expenses in implementing the intervention and whether these costs are included. If the intervention is inexpensive, such as a social media information campaign encouraging the public to get tested for COVID-19, the total cost of the study may be low. Costs can also be distributed across multiple budgets, which can mean that specific project budgets do not cover all the necessary expenses for conducting a trial. For example, research and development funds may be limited to covering research-related costs but not expenses related to the implementation or training of those delivering the service being researched. In comparison, many quasi-experimental studies can be conducted quickly and relatively inexpensively if the necessary data are available.

### Main opportunities

#### Clear ethical and legal framework

The legal tensions and ethical considerations highlighted by the respondents underscore the need to clarify the legal and ethical basis for assessing the appropriateness of time-limited differential exposure to public policy interventions. Currently, the assessment of relatively similar interventions is governed by various legal provisions, depending especially on whether the planned study qualifies as health research.

Thus, there is inconsistency in judging which public policy interventions and programs can be evaluated across different sectors, especially with respect to the requirements for informed consent and determining the legality of implementing differential roll-outs.

Several respondents suggested that legal frameworks, including laws, regulations, and circulars, along with unified interpretation of these, could help guide when differential treatment is deemed permissible. An example where the clarification of the legal foundation was central is a RCT on the effectiveness of incentives for professional development for skilled workers, initiated by the Directorate for Higher Education and Competence on behalf of the Ministry of Education (Box 1).

Box 1. Clarifying the legal basis for differential rollout of an incentive scheme for professional development The White Paper “The Skills Reform—Lifelong Learning” released in 2019 by the Norwegian government emphasized the importance of life-long learning to adapt to labour market and societal changes. Economic incentives were proposed as a measure to enhance professional development of workers. However, due to uncertainty about the benefits of public subsidies for professional and skills development, trials of incentive schemes for professional development was prioritized as one of the three areas of the Skills Program, managed by the Directorate for Higher Education and Skills to achieve the reform’s goals.

A trial was planned to assess the effects of an economic incentive on enrolment in professional development or higher education. A key question was whether the trial’s design might restrict equal access to educational opportunities and thereby violate people’s legal rights under the Student Financial Aid Act, since it involved a control group not receiving the incentive. An independent legal assessment concluded that treating intervention and control groups differently does not violate the law. The key arguments were that the trial in the short term would involve expansion of educational opportunities that otherwise would not be accessible and that in the long term the evidence generated from the trial could promote the Education Act’s goal of providing equal educational opportunities for everyone [21].

#### Strengthening bureaucratic competence, improving political acceptance and fostering support from service providers

The mapping identified several examples of how close collaboration between service delivery entities (like schools or health centres), government departments and researchers from universities, colleges and independent research institutes was crucial for specifying knowledge needs and enabling policy experiments. For example, a close dialogue and transparent processes led by the Ministry of Education and Research and the Directorate for Education and Training were central when random allocation was used to disburse state grants for equipment in schools for vocational and technical education, thereby enabling randomized evaluation of impact. Another example is when taking the time to address concerns from key stakeholders allowed for an RCT to examine the effects of extending the operating hours of Norway’s state monopoly on wine and spirits (Vinmonopolet) [22] (Box 2).

Interventions implemented by government authorities can affect various systems and services. Respondents described how active participation of those affected, such as in schools, healthcare facilities or social security offices and the public administration of municipalities, is essential to understand evidence needs, to develop the intervention and to create acceptance for randomization or, if applicable, quasi-experiments as an approach to acquiring knowledge. Substantial stakeholder involvement is also crucial to ensure that a study does not impose an undue burden on the day-to-day services and routines involved.

Box 2. Generating acceptance for randomized evaluation of extended opening hours of alcohol sales The Norwegian Parliament passed legislation in June 2020 to allow an extra hour of opening time for Vinmonopolet (the state-owned alcoholic beverage retailer) on Saturdays. A year prior, the Ministry of Health and Care Services had commissioned the NIPH to evaluate the effects of extended opening hours if the Parliament approved the legislation. The NIPH research team recommended a randomized roll-out as the preferred evaluation approach. Close engagement with Vinmonopolet and careful consideration of their key concerns were essential prerequisites for their acceptance of the proposed research design.

Vinmonopolet’s main concerns were potential dissatisfaction among customers in control areas who would experience extended opening hours at a later stage and worries about reputation and sales. The NIPH research team had several meetings with Vinmonopolet’s working group to explain possible RCT designs and understand their concerns. Vinmonopolet shared detailed sales data used for power calculations and informing other parts of the design, including the clustering of stores.

The need to find a solution for the RCT became urgent in April 2020 when it became clear that the legislative proposal would likely be adopted, and the extension of opening hours would be implemented shortly thereafter. In subsequent meetings, the NIPH research team presented a menu of five options for how the implementation of extended opening hours could be done. They also presented pros and cons from the Vinmonopolet’s perspective and a research perspective. Agreement was reached to choose cluster-RCT at the trading district level in three phases, excluding major cities from the randomization. Vinmonopolet itself has conveyed six reasons why they ultimately agreed to implement extended opening hours gradually and randomly:

- A general desire to contribute to evidence-based alcohol policy
- A willingness to meet the health authorities’ request for impact evaluation
- The perception of having a good dialogue with the research team and that Vinmonopolet’s concerns were understood and influenced the research design, including the exclusion of major cities from randomization
- The proposed block randomization strategy appeared practically feasible and made it easier for Vinmonopolet to communicate the changes
- Vinmonopolet’s own working group was broadly composed and had a clear mandate to find a practical and feasible solution
- The working group’s proposal was supported by top management and labour unions

#### Building culture and strengthening competence

The mapping highlights the need to cultivate a culture that supports robust impact evaluations. This entails fostering collaboration, particularly between those involved with policy development and policy implementation and those with expertise in research methodologies. Respondents provided examples underscoring how investing in capacity-building initiatives can contribute to an increase in both the quantity and quality of impact evaluations. For example, Norad has organized “research incubators” on impact evaluations, with active participation from civil society organizations operating in development assistance. The Norwegian Tax Administration made a strategic decision in 2010 to establish an analytical environment, which now comprises more than 80 analysts covering core areas of expertise needed to conduct randomized and quasi-experimental studies. During the COVID-19 pandemic, the Norwegian Institute of Public Health established the Centre for Epidemic Interventions Research (CEIR), dedicated to rigorously evaluating public health and social measures for infection control with RCTs and quasi-experimental methods.

#### Improved utilization of existing data and development of new data sources

Easier access to national registry data and better linkage of various data sources can significantly lower the threshold for conducting RCTs and quasi-experimental studies by government agencies. Registry data and surveys are the most used data sources in RCTs and quasi-experimental studies. However, respondents cite access to and linkage of registry data as prominent challenges. For example, robust quasi-experimental studies require detailed data and extensive data linkages. Many such studies are hindered by the time-consuming and challenging process of gaining access to data, often due to privacy concerns and considerations of whether data can be used without consent. When surveys are used, achieving a sufficiently high response rate is a major challenge.

In some sectors, the absence of comparable and precise outcome measures represents a key challenge. In the education sector, the Directorate of Education reported the lack of frequency and continuity in measurements, collecting data for only certain aspects of educational activities, and data sources not being designed for research purposes, as central challenges. For example, the annual student survey and national tests are designed for other purposes than research and are deemed to not be sensitive enough to serve as reliable metrics of learning effects, learning losses, changes in the learning environment or mental health in impact studies.

#### Financing and long-term research programs

Randomized studies often require funding over several years. If the entire process, from introducing the intervention to evaluating measurable outcomes, spans multiple parliamentary periods, long-term financing will often require political cross-party consensus. With political support in place, larger investments, such as long-term research programs, can play a crucial role in facilitating impact evaluations. In the Norwegian context, a concrete example is the LÆREEFFEKT program that was administered by the Research Council of Norway and financed RCTs to study the effects of increased pupil-teacher ratio and small group instruction on pupils’ learning [17, 23]. Furthermore, randomized studies can be integrated into programs designed to follow up on government policies and reforms. An example is the above-mentioned Skills Program managed by the Directorate for Higher Education and Skills (Box 2).

## Discussion

Globally, greater attention is given to strengthening capabilities for generating rigorous evidence on public policy interventions during pandemics, especially public health and social measures. The World Health Organization had, many years prior to the COVID-19 pandemic, advanced a scientific framework for epidemic and pandemic research preparedness [24]. This framework primarily focussed on research to develop new drugs, vaccines and diagnostic tools, paying less attention to the capabilities required to inform a broader, population-level societal response with evidence. Following the COVID-19 pandemic, attention has increased on public health and social measures, which WHO has included among benchmarks to strengthen health emergency capacities and is promoting a global research agenda for [25, 26]. To turn these global aspirations into rigorous evidence on the ground, national government agencies across various sectors are crucial to developing a robust scientific response to pandemics and other public health crises.

Our mapping of Norwegian government agencies’ experiences with RCTs and quasi-experimental evaluations aimed to identify lessons for strengthening the use of these approaches. The survey responses identified factors internal and external to government agencies that need strengthening to enhance research preparedness for new public health crises. Internally, research capabilities can be strengthened by developing robust methodological expertise to determine when RCTs or quasi-experimental evaluations are suitable, and by improving collaboration between government agencies and academic institutions, such as universities and other research institutes. This would facilitate faster mobilization of research resources and expertise when emergencies occur. Crucially, it is important to have adequate exposure to these methods during non-crisis periods, so they are seen as viable options during crises.

Externally, two major factors stood out. First, the importance of having clarity about the legal–ethical framework for allowing differential exposure of the public to policy interventions, which is essential for enabling RCTs or quasi-experimental evaluations. If the legal and ethical pathways are unclear during non-crisis times, they are likely to become significant barriers during crises when time is limited and public acceptance of experimentation is likely to be lower. In Norway, policy experiments, or the lack thereof, was subject to intense scrutiny during and after the COVID-19 pandemic, with the government commissioning several reports about this topic. Several of the legal and ethical challenges raised by our mapping were highlighted in the report “Legal and Ethical Issues Related to the Collection, Accessibility, Sharing, and Use of Data, as well as the Use of Randomized Trials in Crises” [12]. Concrete suggestions included clarifying legal provisions for conducting government-led trials; strengthening the legal basis for the use and sharing of data to reduce uncertainty and delay; and amending the Health Research Act to allow the Regional Committees for Medical and Health Research Ethics to waive the consent requirements when specific conditions are met.

The requirement for informed consent is an especially pronounced issue for conducting policy experiments in Norway, especially when health outcomes are involved. This includes consent to be exposed to the study intervention and to access personal data from administrative records, which often renders RCTs infeasible for public policies. When to exempt such trials from the consent requirement has been widely discussed in the literature [27–29]. The strict informed consent requirement came under scrutiny in Norway during the pandemic, with several research protocols being rejected for exemption. The examples identified in this mapping indicate that for interventions with comparable burdens on participants, such as the risk of adverse consequences and disruption to regular routines, the practice of the informed consent requirement can vary greatly, primarily depending on whether health outcomes are measured. A recent international consensus statement on the ethical design and conduct of cluster-RCTs argues for research ethics committees to consider exempting such trials from informed consent requirements under two conditions: (1) when the research is not feasible without a waiver or modified consent and (2) when the study interventions and data collection procedures involve minimal risk [30]. There is also a growing body of literature exploring the ethics specific to policy experiments [3, 31–34]. For example, Mackay and Chakrabarti argue that it should be ethically defensible for governments to authorize a policy experiment if two conditions are met: (1) the government authority has a right to rule over the policy targeted by the research, meaning that this is a public policy intervention the government has the authority to implement universally and (2) autonomy rights of the participants are not violated by the experiment’s data collection procedures [34]. Recognizing that the strict informed consent requirement in the law hindered important policy experiments from being conducted to generate evidence during the COVID-19 pandemic, the Ministry of Health and Care Services in Norway has recently proposed a new section to the Health Research Act that will allow for exemption from the informed consent requirement if three criteria are fulfilled [35]: (a) the research involves no or minimal risk or inconvenience for the participants, (b) it is difficult or impossible to obtain informed consent from a sufficient sample of the group and (c) the research is expected to have significant benefits for society. At the time of writing, the proposed legislation is undergoing public hearing.

Political acceptance is perhaps the external factor that most significantly influences whether rigorous evaluations of policy interventions are feasible. The political barriers seem to stem from two reasons. First, a preference among policymakers for implementing programs at scale to demonstrate their capacity for action. Government agencies across the board experienced that it is easier to roll out programs with uncertain effects on a large scale than to gradually introduce policies so that these can be researched using RCTs or quasi-experimental methods. Second, political concerns over general hesitancy among the public to accept randomization as an evaluation strategy. While it is difficult to measure the extent of resistance to randomization among the public, results from questionnaire surveys suggest a general aversion to randomization: even in a situation where intervention B is considered inferior to intervention A, many prefer that B be offered to everyone, rather than randomizing between A and B [36]. However, the generalizability of these findings has been questioned and may not be transferable between settings [37]. Gaining political acceptance for randomized evaluation can be particularly challenging during crises, when the public expects swift action and may find it difficult to accept or understand differential exposure to public policy interventions.

Our survey identified several examples of how close dialogue between researchers, implementers, ministries and political leadership has facilitated impact evaluations with randomized or quasi-experimental approaches, from which lessons can be drawn. Political and bureaucratic acceptance at different levels of government may be encouraged by raising awareness of how randomized evaluations can smoothly integrate into a program’s natural implementation process. Among the most prominent actors in the field of policy experiments is J-PAL. Their 2017 report on implementing randomized evaluations at the state and local level in the United States showcase how public policy interventions typically presents opportunities for roll-out that enable rigorous evaluation, yet these opportunities are underutilized [13]. Examples include using randomization to allocate incentives or benefits when demand exceeds availability; leveraging the natural phasing of a program by randomizing its roll-out to municipalities or other implementing units; or exploiting random variation below or above an eligibility threshold. Low-uptake programs with poor evidence base could also benefit; evaluations could be done by randomly exposing individuals or groups to interventions that enhance participation [13].

The respondents in this mapping pointed out that for many interventions, it is more feasible to conduct a quasi-experimental evaluation than a RCT, and these methods should be considered as complementary approaches. Typically, quasi-experimental studies are conducted after researchers have identified variations in how an intervention was introduced and rolled out, for example, when an intervention is implemented at different times in various schools, primary care offices, social service offices or other units. However, it is also possible and usually preferable to plan for evaluation in advance by implementing the intervention in a way that allows for studying its effects. This requires close collaboration between decision-makers and researchers with relevant methodological expertise. For example, when door-to-door testing was rolled out at a district level in Norway during the COVID-19 pandemic, the local administration carried out the intervention at the basic statistical level unit, which allowed researchers to measure the effect on testing rates at this level [38]. However, a major issue with quasi-experimental evaluations is that these studies can lead to unreliable inferences about effects if the key assumptions of the methods are not met. This issue was particularly evident during the COVID-19 pandemic, when numerous quasi-experimental evaluations purported to demonstrate the impact of various public health and social measures, such as a heavily criticized study on the effects of mask mandates published in Proceedings of the National Academy of Sciences [39, 40]. Thus, the majority of these studies did not meet the methodological standards necessary for drawing valid conclusions [9].

Principles for crisis management during epidemics and pandemics prescribe prompt action; under these circumstances, decision-makers must balance the use of reliable evidence with the timeliness required to respond to immediate threats, respond to public opinion, act pre-cautionary and accept that evidence guiding action may carry greater uncertainty and be less reliable [41–43]. In many circumstances, especially during the early phase, decision-makers will have to proceed without the guidance of high-certainty evidence, such as those generated by RCTs or high-quality quasi-experimental evaluations. However, as experience from COVID-19 showed, a public health crisis reaches a stage where balance need to be struck between strict interventions that contain transmission and less intrusive interventions that allow for societal activities and minimize negative consequences. In this situation, policymakers will face “equipoise” – namely, that two or more alternatives carry similar uncertainty about their beneficial effects on transmission and the burden of negative consequences [33]. During these circumstances, rolling out interventions differentially over a relatively short period could help provide the evidence that decision-makers need to manage a pandemic with interventions with a better balance between benefits and harms. To align evidence-generation processes with the urgency of a pandemic, opportunities for enabling faster evaluations, as identified by this mapping, must be pursued in advance of the next public health crises. Key steps include clear legal and ethical pathways, including exemption from informed consent for population-level interventions; infrastructure for timely data collection, such as high-quality registry data; public debate and education about the benefits and harms of generating evidence through trials to achieve greater public acceptance for participating in such trials; and having expertise and capacity for conducting such evaluations. A recent summary of barriers to RCTs in Norway during the COVID-19 pandemic identified several barriers that, if addressed, could enable faster impact evaluations during a pandemic [44].

Crisis management principles on sectoral responsibility and collaboration can guide how sectors should share responsibility for strengthening research preparedness for crises. In Norway, four crisis management principles – responsibility, conformity, subsidiarity and cooperation – are central to Norway’s approach to emergency preparedness [45] and align with those of other countries [46, 47], especially the Nordics [48, 49]. The responsibility principle assigns crisis responsibility to the organization that manages the area under non-crisis conditions. The conformity principle ensures that the crisis structure remains similar to the usual organizational structure. The subsidiarity principle mandates that crises be handled at the lowest possible level. Finally, the cooperation principle emphasizes that authorities and agencies must independently ensure effective collaboration with relevant partners. Together, these principles serve to ensure that institutions can apply strengths from their day-to-day responsibilities in non-crisis times to a crisis situation, such as a pandemic. Embedded in this approach to crisis preparedness is an expectation that actions are knowledge-based, involving the generation and use of research to guide decisions. A key implication is that building capacity for evidence generation before the crisis strikes needs to happen in the institutions that have the day-to-day responsibilities for sectors that may be affected by a pandemic. For example, during COVID-19, many public health and social measures were implemented in the educational sector. Building awareness of and capabilities for RCTs and quasi-experimental evaluations in sectors outside health can therefore contribute to stronger research preparedness before crises.

Our mapping primarily explored how government agencies generate evidence with RCTs and quasi-experimental methods and not how the evidence from these evaluations were used to inform policy. Since these agencies are closer to policymaking, these may be better positioned than others – such as universities – to bring evidence from impact evaluations to the attention of policymakers. However, literature on evidence-informed policymaking emphasizes that policymakers rely on diverse sources of knowledge, including public opinion, personal beliefs and values, and that evidence on effects from RCTs or quasi-experimental evaluations rarely are the sole source of evidence [50–52]. This might limit the extent to which evidence from these methods can actually influence policy. Indeed, our study came across several examples underscoring this point. For example, we identified that politicians introduced a new teacher density reform in primary schools before a RCT evaluating the effects of this measure had been completed [53]. During a pandemic, many interventions involved a large burden on personal freedoms and economic consequences. Policymakers might be incentivized to be guided by evidence from RCTs and quasi-experiments if such evaluations can capture the trade-off between the benefits for controlling transmissions and the burden on individuals and society. Dedicated efforts to choose the least intrusive but effective measure can perhaps also contribute to greater public support for impact evaluations. Accordingly, more systematic approaches to evidence generation with RCTs and quasi-experiments within government agencies – including collaboration between politicians, policymakers, researchers and the public to identify valued outcomes – could support both the production and use of such evidence.

We did not identify similar mapping from other countries. However, several major research institutions, such as J-PAL, have actively formed collaborations with national and local governments to undertake RCTs and quasi-experimental approaches, especially in the development sector [1]. Within government, an example of efforts to enhance rigorous evaluations of public policy is found in the United Kingdom, where the “What Works Initiative” was established in 2013 [54]. As part of the initiative’s commitment to promoting evidence-informed policy, the United Kingdom government created a network of research centres dedicated to supporting decision-makers with knowledge about the effects of interventions in areas such as health, education and criminal justice. An interesting mechanism for competence building about evaluation methods in the public sector is the Evaluation and Trial Advice Panel, which was established to offer civil service guidance and training in impact evaluation methods. Since 2015, the panel has offered guidance for more than 170 projects, spanning areas such as education, health, transportation, crime and more [55].

## Conclusions

This analysis of Norwegian government agencies’ use of randomized trials and quasi-experimental evaluations underscores the legal, ethical, political and practical considerations that require attention to enable greater use of these methods for evaluating public policy interventions and for strengthening research capabilities that can be deployed during public health crises. Greater political acceptance for systematic, gradual – and if possible, randomized – implementation of reforms and measures could facilitate improved learning about the effects of interventions. This could lead to more efficient and targeted policy development, increasing the chances that effective interventions are scaled up, or that ineffective interventions are deprioritized, both during public health crises and in non-crisis periods.

## Supplementary Information

Supplementary Material 1

## Data Availability

The survey responses, collected as part of a government-commissioned mapping, are considered government documents and are accessible upon request under Act relating to the right of access to documents held by public authorities and public undertakings (Freedom of Information Act). An abridged summary of each government agency’s response to the survey is included as an appendix in this report, available in Norwegian: https://www.fhi.no/publ/2023/randomiserte-og-kvasieksperimentelle-studier-i-den-statlige-forvaltningen-en-kartlegging/.

## Abbreviations

J-PAL: Abdul Latif Jameel Poverty Action Lab
NIPH: Norwegian Institute of Public Health
RCT: Randomized controlled trial
