# Supplementary material for: Strengthening research preparedness for crises: lessons from Norwegian government agencies in using randomized trials and quasi-experimental methods to evaluate public policy interventions
Source: Health Res Policy Syst. 2025 Jan 13;23:8. doi: 10.1186/s12961-024-01271-y (PMC11726906; doi:10.1186/s12961-024-01271-y)
Supplement: Supplementary file 1 — Supplementary Material 1 [file 12961_2024_1271_MOESM1_ESM.docx]

**Appendix 1**

**Survey: Mapping experiences with randomized trials and quasi-experimental methods**

**Part 1: Introduction and definitions**

Through the allocation letter, the Norwegian Institute of Public Health (NIPH) has been assigned the responsibility to coordinate a survey on how governmental agencies use randomized trials and quasi-experimental methods to inform public policy with evidence. We're interested in experiences from evaluations the agency has conducted on its own, commissioned to researchers, or been actively involved with in other ways. The objective of the survey is to explore the agencies' experiences with such methods, the strengths, weaknesses, and limitations of using these methods, and what prevents and promotes their use.

The assignment text in the allocation letter described the NIPH, the Directorate for Children, Youth and Family Affairs, the Norwegian Labour and Welfare Administration, the Directorate for Education and Training, the Norwegian Tax Administration, and the Norwegian Agency for Development Cooperation (Norad) to be among the most relevant agencies for such an assignment, but that other state and municipal agencies may also be invited. NIPH is assigned the responsibility to coordinate the execution of this survey and to present the results in a report by May 1, 2023.

**Key definitions**

1. **Public policy interventions**

Government or municipal departments can use a variety of interventions to implement public policy. Such interventions include:

- legal interventions, for example new laws and regulations
- economic interventions, for examples changes in taxes and duties
- organizational interventions, for example reorganization, centralization, and decentralization of government functions
- educational interventions, for example guidelines, competence development initiatives, or information campaigns

These interventions, on a larger or smaller scale, can be the basis for effect evaluation with randomized trials or quasi-experimental studies.

1. **Randomized trials**

In randomized trials, the effect of an intervention is measured by comparing participants who are randomly allocated to be exposed or not exposed to the intervention. Randomization can also occur through the gradual and random phase-in of an intervention. The participants can be individuals or groups of individuals, e.g., school classes, schools, or municipalities. The latter is usually called a cluster-randomized trial. Randomizing a sufficient number of participants/clusters makes it probable that the intervention group and the control group are similar with respect to characteristics that can influence the outcome, so that any differences in the outcome can be attributed to the intervention being implemented.

1. **Quasi-experimental studies**

In quasi-experimental studies, there has not been a random allocation to intervention or control groups. However, causal relationships can still be uncovered because the distribution of individuals or clusters to an intervention has occurred in such a way that the groups are comparable. The degree of comparability will vary between different types of quasi-experimental studies.

**Part 2: Use of randomized trials to evaluate effects**

With the questions in part 2, we aim to map experiences with randomized trials that the agency has conducted itself, has commissioned through assignments to researchers, or in other ways have facilitated (e.g., as an active part in a collaboration with researchers).

**Scale**

1. Approximately, how many randomized trials has the agency conducted—either with its own capacity, by commissioning, and/or by being an active participant in a collaboration with researchers—over the last five years?

| - None | - 1-5 | - 6-15 | - >15 | - Don’t know |
| --- | --- | --- | --- | --- |

1. Approximately, what percentage of these were conducted by others (e.g., researchers from the institute sector, universities, colleges) by commissioning? Specify in percentage.
2. Do you have examples of interventions that have been evaluated with randomized trials?

Describe

- The target group that was the focus of the trial
- The intervention/measure being tested
- Desired/undesired results
- Overall lessons learned from the study and the process

1. How many randomized studies have been attempted to be planned, but stopped due to various types of challenges? (e.g., ethical, legal, political, other). Please provide examples of specific studies that have been stopped and the challenges that were encountered.
2. What do you consider the advantages of using randomized trials?
3. What do you consider to be methodological challenges in using randomized trials?
4. In what ways has the agency attempted to facilitate the evaluation of an intervention with randomized trials?

**Quality and data sources**

1. What is the agency doing to ensure that randomized trials are conducted in accordance with scientific principles and can generate credible answers? Examples could include: ensuring that the studies have sufficient statistical power, the optimal randomization technique is chosen, etc.
2. What data sources (e.g., health registers or surveys) have been used to conduct randomized trials?
3. What challenges have the agency or researchers engaged by the agency encountered in connection with the use of such data sources?

**Challenges and opportunities**

1. What legal basis and regulations does the agency use to justify the conduct of randomized trials?

For example, §12 of the Labour Market Act opens up for experiments: "The Department may issue regulations concerning experiments with labor market measures."

1. What legal challenges have statutory provisions and regulations created for the conduct of randomized trials?
2. What ethical challenges have the agency or researchers the agency collaborates with encountered in connection with the planning and conduct of randomized trials?
3. What political challenges have the agency or researchers the agency collaborates with encountered in connection with the planning and conduct of randomized trials? An example of a political challenge is that the time window for implementing and rolling out a measure on a large scale does not necessarily match the time needed to conduct a thorough effect evaluation.
4. Beyond ethical, legal, and political challenges, what other factors hinder the widespread use of randomized trials to evaluate measures recommended and implemented by the agency?
5. What can facilitate increased use of randomized trials to evaluate the agency's use of measures?
6. Which interventions within the agency's sectoral responsibility are suitable for evaluation with randomized trials?
7. Which interventions within the agency's sectoral responsibility are not suitable for evaluation with randomized trials?

**Part 3: Use of quasi-experimental methods to evaluate effects**

With the questions in part 3, we aim to map experiences with quasi-experimental studies that the agency has conducted itself, has commissioned through assignments to researchers, or in other ways have facilitated.

1. Approximately, how many quasi-experimental studies has the agency conducted—either with its own capacity, by commissioning, or in other ways collaborating with researchers—in the last five years?

| - None | - 1-5 | - 6-15 | - >15 | - Don’t know |
| --- | --- | --- | --- | --- |

1. Approximately, what percentage of these were conducted by others (e.g., researchers from the institute sector, universities, colleges) by commissioning? Specify in percent.
2. Can you provide examples of interventions that have been evaluated with quasi-experimental methods?
3. Do you have examples of quasi-experimental studies that have been attempted to be planned but not completed due to various types of challenges? (e.g., ethical, legal, lack of data, the intervention not implemented as planned). Please provide examples of specific studies that have been stopped and the challenges that were encountered.
4. In what ways has the agency attempted to facilitate the evaluation of an intervention with quasi-experimental methods?
5. What does the agency consider to be the advantages of conducting quasi-experimental studies?
6. What does the agency consider to be the methodological challenges of conducting quasi-experimental studies?

**Quality and data sources**

1. What does the agency do to ensure that quasi-experimental studies are conducted in accordance with scientific principles and can provide credible answers? Examples may include: encouraging practices such as preregistration; courses and other measures for competence development.
2. Which data sources (e.g., health registries or surveys) have been used for quasi-experimental studies conducted with own capacity and/or through procurement?
3. What challenges have the agency or researchers engaged by the agency encountered in connection with the use of such data sources?

**Challenges and opportunities**

1. What legal challenges have statutory provisions and regulations created for conducting quasi-experimental studies?
2. What ethical challenges have the agency or researchers collaborating with the agency encountered in relation to the planning and implementation of quasi-experimental studies?
3. What political challenges have the agency or researchers collaborating with the agency encountered in relation to the planning and implementation of quasi-experimental controlled studies? An example of a political challenge is that the time window for implementing and rolling out an intervention on a large scale does not necessarily match the time needed to conduct a thorough effectiveness evaluation.
4. Beyond ethical, legal, and political challenges, what other factors hinder the widespread use of quasi-experimental studies to evaluate interventions recommended and implemented by the agency?
5. Describe the factors that can facilitate increased use of quasi-experimental studies for evaluating the agency's intervention use?
6. Which interventions within the agency's sectoral responsibility are suitable for evaluation with quasi-experimental studies?
7. Which interventions within the agency's sectoral responsibility are not suitable for evaluation with quasi-experimental studies?

**Part 4: General questions about capacity and pre-requisites for conducting effect evaluations**

In this last section, we ask general questions about the agency's prerequisites for promoting the use of randomized trials and quasi-experimental methods—hereafter referred to as impact evaluations.

1. How many work with conducting and/or commissioning impact evaluations?

| - None | - 1-5 | - 6-15 | - >15 |
| --- | --- | --- | --- |

1. What expertise does the agency need more of in order to conduct and/or collaborate with external researchers and achieve more impact evaluations?
2. Could you explain how the responsibilities and organizational roles are divided between those involved in developing, recommending, and implementing policies or programs, and those responsible for commissioning impact evaluations?
3. Which publication channels (e.g., internal reports, peer-reviewed journals, externally available reports, others) are used to disseminate completed impact evaluations? Please feel free to share links to published reports and articles.
4. Are there any other experiences the agency would like to add, which were not covered by the questions in parts 2-4?
